# Combining emotion regulation and mindfulness skills for preventing depression relapse: a randomized-controlled study

**DOI:** 10.1186/s40479-017-0064-6

**Published:** 2017-07-05

**Authors:** Matilde Elices, Joaquim Soler, Albert Feliu-Soler, Cristina Carmona, Thais Tiana, Juan C. Pascual, Azucena García-Palacios, Enric Álvarez

**Affiliations:** 1 Servei de Psiquiatria, Hospital de la Santa Creu i Sant Pau (Barcelona), Av. Sant Antoni Mª Claret 167, 08025 Barcelona, Spain; 2 Centro de Investigación Biomédica en Red de Salud Mental (CIBERSAM), Institut d’Investigació Biomèdica–Sant Pau (IIB-Sant Pau), Barcelona, Spain; 3 Departament de Psiquiatria i Medicina Legal, Universitat Autònoma de Barcelona, Barcelona, Spain; 4 Programa de Cognición, Instituto de Fundamentos y Métodos en Psicología, Facultad de Psicología, Universidad de la República, Montevideo, Uruguay; 5 Departament de Psicologia Clínica i de la Salut, Universitat Autònoma de Barcelona, Barcelona, Spain; 6 Institut de Recerca Sant Joan de Déu, Esplugues de Llobregat, Spain. Primary Care Prevention and Health Promotion Research Network, RedIAPP, Madrid, Spain; 7 Universitat Jaume I, Castellón, Spain; 8 CIBER Fisiopatología Obesidad y Nutrición (CIBERObn), Instituto Salud Carlos III, Madrid, Spain

**Keywords:** Mindfulness, Emotion regulation, Major depression, Relapse, Recurrence

## Abstract

**Background:**

Dialectical behavioral therapy (DBT) skills have become increasingly used to treat several psychiatric conditions, including major depressive disorder (MDD). The aim of the study was to investigate the efficacy of an intervention that combines emotion regulation and mindfulness skills of DBT to prevent depression relapse/recurrence.

**Methods:**

A total of 75 individuals (79% females; mean age, 52 years) with a diagnosis of MDD in complete or partial remission were recruited. Participants were randomly allocated either to an intervention combining emotion regulation and mindfulness skills (ER + M group, *n* = 37) or to a psychoeducative program (*n* = 38). After the 10-week treatment period, participants were followed for 1 year. Analyses were run in per-protocol (PP) and intention-to-treat (ITT) samples. The primary outcome measure was time to depression relapse/recurrence.

**Results:**

ER + M training was not more effective than the control intervention in preventing depression relapse. However, PP and ITT analyses showed that participants trained in ER + M presented a significant reduction in depressive symptoms and overall psychopathology. Based on the PP and ITT analyses, neither of the interventions were related with an increase in dispositional mindfulness.

**Conclusions:**

More studies are needed to confirm the efficacy of ER + M to decrease depressive symptoms and overall psychopathology.

**Trial registration:**

NCT02747134. Registered on 20 April 2016.

## Background

Major Depressive Disorder (MDD) is one of the most prevalent and disabling mental disorders, and it is frequently associated with a high risk of suicide [1]. The World Health Organization estimates that, by the year 2030, depression will be the leading cause of disease burden globally [2]. This disorder has a highly recurrent nature and a poor long-term prognosis because the probability of another depressive episode increases with each relapse/recurrence. Available studies have reported relapse rates ranging from 50 to 90%, and these data suggest that a patient diagnosed with MDD will experience an average of four episodes during his/her lifetime [3]. These high relapse rates require effective treatments, not only to manage depressive symptoms in the acute phase, but especially to develop effective interventions to prevent relapse and recurrence.

Dialectical Behavioral Therapy (DBT) is a multi-component treatment initially developed for patients with borderline personality disorder [4]. Because DBT is based on a skills deficit model, the treatment focuses on skills training. In standard DBT, skills are organized into four modules: mindfulness, interpersonal effectiveness, emotion regulation, and distress tolerance [5]. In recent years, DBT—particularly, DBT skill training—has been used to treat diverse populations affected by emotion dysregulation [6]. In subjects with an MDD diagnosis, some studies have compared the effects of DBT skills training to antidepressant medications (AM). The first study to perform this comparison [7] found no significant differences between the interventions in terms of improvement in depression symptoms or post-treatment remission rates (28 weeks). However, that study did find that remission rates were more favorable in the DBT group (75% vs. 31%) after 6 months of follow up [7]. These findings were subsequently confirmed in another study [8] in which a significantly higher number of patients receiving DBT + AM achieved remission (both at post-treatment and follow-up) compared to AM alone. Findings from a pilot study conducted by Harley et al. showed that skills training was more efficacious than treatment as usual (TAU) to decrease depression in individuals with treatment-resistant MDD [9].

One of the major advantages of DBT skill training is that clinicians can combine these skills in different ways to target specific clinical symptoms. This allows implementation of DBT skill training in settings where delivering the complete DBT treatment package would not be feasible. Furthermore, recent research suggests that some skills could be more closely related to positive clinical outcomes than others [10, 11]. Among the various DBT skills, we hypothesize that emotion regulation (ER) and mindfulness (M) skills may be specifically beneficial for patients with MDD diagnosis. ER refers to the set of skills used for understanding and learning to regulate emotions by 1) attending to emotional vulnerability to cues, 2) managing attention and appraisal of the cues, 3) changing physiological, cognitive, and experiential responses and action urges, and 4) dealing with the consequences of the emotional response [12]. The successful application of ER skills has been associated with a significant decrease in the severity of depressive symptoms [13, 14]. For instance, Berking and colleagues [15] assessed participants with MDD criteria enrolled in a treatment program consisting of CBT + ER training, finding that these patients experienced a greater reduction in depression and negative affect as well as a larger increase of well-being, compared to those receiving only CBT. In DBT, ER skills training is oriented to encouraging behavioral activation (BA) by training patients in “opposite action” (OA) to depressive symptoms. OA training aims to target avoidance by changing expressions (facial and postural) and behavior in a way that is opposite to feelings of sadness. DBT also includes skills training to help subjects obtain positive emotions in both the short and long-term by increasing daily pleasant events and by fostering values-related activities [6].

Like DBT, there are other interventions that contain mindfulness training, including Mindfulness-Based Cognitive Therapy (MBCT) [16], which is also focused on increasing acceptance and ER. Research on MBCT suggests that increasing mindfulness skills could be crucial to prevent MDD relapse by helping patients to identify and disengage from cognitive non-adaptive patterns, thus avoiding the re-establishment of a depressive episode [17–20].

In this context, we designed the present randomized controlled study to investigate the efficacy of an intervention that combines ER with mindfulness (ER + M) skills to reduce rates of depressive relapse/recurrence. Participants with a previous diagnosis of MDD in partial or total remission were considered eligible to participate. A second aim was to assess the effects of the intervention on depressive symptoms, general psychiatric symptoms, and mindfulness capacities.

## Methods

### Participants and recruitment

A total of 110 patients were assessed for eligibility and 75 were included in the study. Participants were recruited from outpatient facilities at the Psychiatric Service of the Hospital de la Santa Creu I Sant Pau and from the Adult Mental Health Center (Barcelona, Spain). Based on effect sizes reported in similar, previous research studies [17, 18] and considering an expected relapse/recurrence rate of 35 and 70%, respectively, in the treatment (ER + M) and control groups (psychoeducation), a minimum sample of 60 participants (30 per group) was required to ensure power of .80 at a significance level of α = .05 [19].

Inclusion criteria were: 1) age 18 to 75 years; 2) previous diagnosis of MDD in complete or partial remission (according to the major depressive section of the Structured Clinical Interview for DSM-IV Axis I Disorders; SCID-I) at study inclusion and scores < 17 on the Hamilton Depression Rating Scale (HDRS); 3) having experienced the last major depressive episode between 18 and 2 months prior to study initiation; 4) at least two previous depressive episodes; 5) pharmacological treatments were permitted provided that the medication(s) remained unchanged for the 2 months before inclusion in the study. Exclusion criteria were: 1) current MDD or any other affective disorder (DSM-IV-TR criteria); 2) alcohol or drug dependence, schizophrenia or psychotic disorders (DSM-IV-TR criteria); 3) personality disorders (DSM-IV-TR criteria); severe physical conditions such as organic brain syndrome, neurological disease; mental retardation or cognitive impairments; 4) current psychological treatment; 5) changes in pharmacological treatment during the study.

An independent statistician randomized the participants who met all inclusion criteria using a computer-generated sequence (blocks of four participants without stratification). The study coordinators informed the patients about their treatment assignment, but the interviewers were blinded to the patient’s condition throughout the study. Participants were instructed not to disclose information about their treatment group to the interviewer.

Participants were informed of the study procedures and required to sign the informed consent form prior to the assessments. Participants received no payments of any kind for taking part in the study. The trial protocol was approved by the ethics committee of the Hospital de la Santa Creu I Sant Pau and was retrospectively registered on clinicaltrials.gov (NCT02747134).

### Study design and procedures

This was a randomized, single blind, two–arm (experimental and control) clinical trial. The study consisted of two phases: phase 1: treatment (10 weeks) and phase II: 1-year (52 week) follow-up period. Primary outcome measures were assessed at three time points during the treatment phase [i.e., pre-intervention, intervention midpoint (5 weeks) and post-intervention] and bi-monthly during the follow-up period. Assessments of secondary outcomes were conducted at only two time points: pre-and-post intervention.

The primary outcome measure was time to relapse/recurrence of major depression, which was defined as meeting SCID-I criteria for at least 2 weeks. In the follow-up assessments, participants were asked to estimate, as precisely as possible, the data of onset of the depressive episode. If the participant could remember the month but not the day of onset, the relapse/recurrence was coded as occurring in the middle of the month (second week). In patients who reported onset at the “beginning of the month”, the relapse/recurrence was encoded as occurring in the first week of the month while “end of the month” was encoded as the fourth week of the month. If a participant presented relapse criteria at the time of the follow up interview, an additional interview was scheduled for the next week to determine if symptoms were present for at least 15 days.

### Treatments

Both interventions were applied in a group format of 8 to 12 participants each. The ER + M treatments consisted of 10 sessions (once weekly, 120 min each). The psychoeducation treatments consisted of five sessions, delivered every 2 weeks (90 min). The treatments in both groups were scheduled this way to ensure that the time frame of both interventions was the same (i.e., 10 weeks).

#### Emotion regulation and mindfulness skills (ER + M)

This intervention was performed in accordance with the principles and structure of DBT skills training. Participants were given step-by-step instructions, rehearsal exercises, and homework assignments to practice the skills learned in the sessions. The first session provided an overview of the training goals and an explanation of the differences between the three states of mind (i.e., emotional mind, rational mind and wise mind). Thereafter, “what” (i.e., describe, observe, participate) and “how” (i.e., without judging, being effective, and one-mindfully) mindfulness skills were presented and practiced during the session. Sessions 3 to 10 began with 10–15 min of mindfulness practice followed by homework review. The focus of these sessions was on behavioral activation skills, including “opposite action”, “accumulating positive emotions” (i.e., increasing daily positive experiences in the short and long term) and “building mastery” (i.e., engaging in activities that foster a sense of self-efficacy and competency) [4]. As with mindfulness skills, participants were instructed to practice the ER exercises during the week and each therapy session began with the review of homework assignments. The content of each session was tailored to participants with MDD. For example, mindfulness practice included examples of how to deal with negative thoughts and emotions (e.g., sadness, guilt, anger) associated with depressive states. Similarly, ER skills were oriented towards fostering “opposite action” to sadness and to increasing the occurrence of positive life-events. Two DBT-certified therapists (therapist and co-therapist) with extensive training in DBT and mindfulness-based interventions conducted the treatment. In order to provide feedback and supervision of the interventions, other team members followed the sessions using a closed-circuit television. Video cameras transmitted the signal but the sessions were not recorded.

#### Psychoeducation

The psycho-educative program aimed at providing general education about MDD, increasing the patient’s knowledge about depression and depression relapse. To this end, participants received evidence-based information about the psychological process of relapse, emphasizing the relevance of recognizing warning signs of depression. A session was dedicated to explaining how healthy habits (i.e., sleeping, diet, and physical exercise) could improve low mood. Finally, skills for managing interpersonal and family conflicts were taught. The intervention had a class format but unlike ER + M, no homework was assigned. A psychologist with training in cognitive-behavioral therapy conducted the control intervention.

See Table 1 for a detailed description of the treatment curricula.

**Table 1:** Treatment curricula for both interventions

Condition	Week	Skills	Target problems with
Emotion Regulation + Mindfulness	1	Wise mind, how and what skills	Rumination, Depression-related cognitions, emotions and physical sensations
	2		
	3		
	4	Understand emotional process: identify and label emotions	Emotional processing
	5		
	6		
	7	Opposite Action	Changing emotional expressions/behaviors
	8		
	9	Accumulate Positives	Reduce vulnerability to depression, increase positive reinforcement, increase self-efficacy
	10	Build Mastery	
Psychoeducation	1	Psychological process of relapse	Increase patients’ knowledge about depression, and the mechanisms involved in depressive relapse
	2	Recognizing warning signs of depression	
	3	How habits affect depression (e.g. sleep, food, physical activity)	
	4		
	5	Managing interpersonal conflict	

### Measures

#### Primary outcome measure

The primary outcome measure was time to relapse/recurrence of major depression, defined as the emergence of a new acute episode according to DSM-IV-TR criteria [21]. The SCID-I was used to assess major depressive criteria.

#### Secondary outcome measures

To assess changes in depressive symptoms during the intervention phase, the Beck Depression Inventory (BDI-II) [22] and the HDRS [23] were used (self-reported and clinician-reported symptoms, respectively). To assess the impact of both interventions on general psychiatric symptoms, the Symptom Checklist 90 (SCL-90) [24] was used. The SCL-90 is a self-reported scale containing 90 items rated on a 5-point Likert scale (0 = not at all, 4 = extremely). The Global Severity Index (GSI) was used as a global measure of psychopathology. Since one of the interventions included mindfulness training, the Mindful Attention Awareness Scale (MAAS) [25] was also used to assess changes in dispositional mindfulness. The MAAS is a unifactorial scale that measures the general tendency to be attentive and aware of the present experience in daily life. Items are rated on a 6-point Likert type scale. Respondents rate how often they are acting on automatic pilot, preoccupied, and not paying attention to the present moment. The MAAS has good internal consistency and adequate convergent, discriminant and incremental validity [25].

### Statistical analysis

Normality tests (skewness, kurtosis and boxplots) were performed to determine if the data had a normal distribution. Outliers were not identified. Skewness and kurtosis values were within the acceptable range of −2.00 to +2.00 and −5.00 and +5.00 respectively [26]: skewness and kurtosis, respectively, ranged from − .17 to .61 and − .57 to .65. A normal distribution was not confirmed for the number of previous episodes (Skewness = 5.54 and Kurtosis = 33.93) and thus, a non-parametric test (Mann-Whitney *U* Test) was run. Descriptive statistics and baseline clinical characteristics were analyzed using either chi-squared test or Fisher’s exact test for categorical variables and *t*-test for continuous ones.

For the time-to-event data (i.e., time to onset of relapse, in weeks), Kaplan-Meier curves were plotted. The treatment condition and the control intervention were compared using long-rank statistics (Mantel-Cox test). Analyses of secondary outcomes (i.e., BDI-II, HDRS, SCL-90-GSI and MAAS) were performed in the per-protocol (PP) population (defined as participants who complete pre-post assessments) and the intended-to-treat (TT) sample. For the completers analyses, participants were included if they attended ≥ 80% of the interventions. For the ITT analyses, we used a standard conservative method in which the participant’s last observation was carried forward (LOCF) to account for missing data [27]. In both analyses (i.e., PP and ITT), analyses of covariance (ANCOVA) were performed to compare post-measurement scores on secondary outcome measures (i.e., BDI-II, HDRS, SCL-90-GSI and MAAS) between the two groups (ER + M vs. PE), controlling for baseline levels. All hypotheses were two-sided and tested at a significance level of *p* < .05. Estimated effect sizes were calculated as Cohen’s d and evaluated as follows: small = 0.20, moderate = 0.50 and large = 0.80 [28]. All analyses were performed with the SPSS 19.0 statistical software package (SPSS-IBM; Chicago, IL, USA).

## Results

### Patient flow and dropout

Of the 110 participants assessed for eligibility, a total of 75 were randomized to ER + M (37 patients) or psychoeducation (38). Reasons for exclusion included: failing to meet inclusion criteria (*n* = 21) and refusing to participate in the study (*n* = 14). The proportion of participants in total (HDRS <8) or partial (HDRS between 8 and 17) remission was similar in both groups [*X* ^2^ (1, *N* = 78) = .65, *p* = .42]. See Table 2.

**Table 2:** Summary of demographics and clinical characteristics at baseline by group

	ER + M (*n* = 37)		PE (*n* = 38)		*X* ^*2/*^ *F*	*t/U*	*p*
	M	SD	M	SD			
Gender (% females)	75.7		81.6		.38		.53
Marital Status (% of married)	58.3		73.3		2.98		.40
Years of education	11.75	5.40	11.35	4.88		.33	.38
Age	51.35	10.73	53.82	10.87		−.98	.33
Number of PDE	2.81	2.51	2.18	.51		626.00	.20
Partial remission (%)	40.5		31.6		.65		.42
Total remission (%)	59.5		68.4				
Pharmacological treatment (%)							
Antidepressants	91.9		97.4		.35		
SSRI (%)	62.2		57.9		.14		.71
TCAs (%)	35.1		34.2		.01		.93
Dual-Action Antidepressants	5.4		5.3		1.00		
Other Antidepressants	13.5		21.1		.54		
Mood Stabilizers	18.9		23.7		.25		.61
Antipsychotics	8.1		13.2		.71		
Benzodiazepines	29.7		36.8		2.21		.51

*ER + M* Emotion Regulation plus Mindfulness, *PE* Psychoeducation, *PDE* Previous Depressive Episodes, *SSRI* Selective serotonin re-uptake inhibitors, *TCAs* Tricyclic antidepressants

During the intervention phase, three participants (8%) dropped out of the treatment group while 10 participants (26%) dropped out of the control group (Fisher’s Exact Test: *p* = .60). Two participants in the ER + M group missed two or more consecutive sessions (without providing any justification for doing so) and were therefore considered to have dropped out of treatment. One participant in the treatment group had to abandon therapy due to incompatibilities with work schedule. Of the 10 subjects who abandoned the PE group, eight missed more than two consecutive sessions and two withdrew due to life events (one participant moved out of the country and one had scheduling incompatibilities with work).

**Fig. 1:**
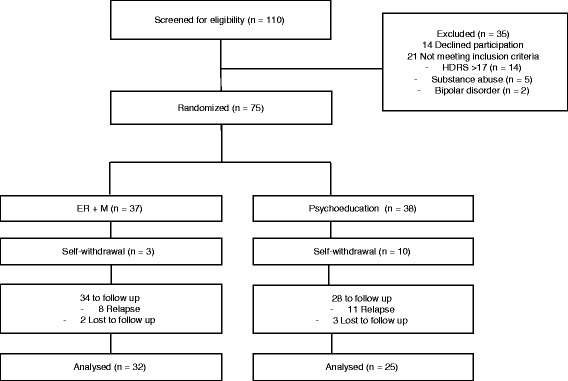
Diagram showing participant’s flow through the study. ER + M = Emotion Regulation plus Mindfulness skills

After accounting for dropouts, 34 and 28 participants, respectively, remained in the treatment and control groups. A total of five patients (two in the treatment group and three in the control group) were lost to follow-up. No significant between-group differences were observed in the number of subjects lost to follow-up (Fisher’s Exact Test: *p* = 0.65) (Fig. 1).

### Demographic and clinical characteristics of the sample

Most (79%) of the participants were women. Mean age was 52 years. No differences were observed between the treatment and control groups at baseline in terms of demographic or clinical characteristics, nor were differences seen in pharmacological treatments. Similarly, there were no differences between the groups in the mean number of previous depressive episodes [*U* (75) = 626.00, *Z* = −1.28, *p* = .20]. Demographic and clinical characteristics are summarized in Table 2.

### Primary outcome: relapse/recurrence to major depression

**Fig. 2:**
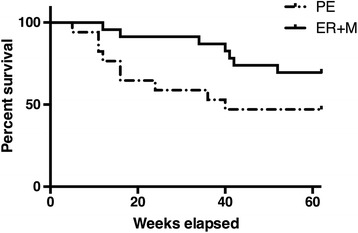
Survival curves comparing relapse to major depression for emotion regulation plus mindfulness skills (ER + M; *n* = 37) and Psychoeducation (PE; *n* = 38)

Figure 2 shows survival (i.e., non-relapse) over the 62-week study period for the two groups. No significant differences between the groups were observed in time to relapse/recurrence (Long Rank Test, *X* ^2^ = 2.83, *p* = .09).

### Secondary outcomes: intervention effects on depression, general psychiatric symptoms, and dispositional mindfulness

ANCOVAs conducted in the per-protocol sample [between-subjects factor: group (ER + M, PE); covariate: baseline scores] revealed a significant main effect of group for HDRS scores *F*(1, 53) = 4.08, *p* = .048] and SCL-90-GSI scores *F*(1, 35) = 4.55, *p* = .04]. These results were maintained in the ITT analyses, HDRS [*F*(1,72) = 4.45, *p* = .04], and SCL-90-GSI [*F*(1,72) = 4.24, *p* = .04]. No statistically significant differences between conditions were observed for BDI [*F*(1,59) = .85, *p* = .36] or MASS scores [*F*(1,39) = 1.05, *p* = .31] at post-intervention in the PP sample. The ANCOVAs performed in the ITT sample also revealed no main effect of group for BDI [*F*(1,72) = .83, *p* = .36 or MASS [*F*(1,72) = .75, *p* = .40] scores. Table 3 shows details of the analyses conducted in the PP sample.

**Table 3:** Analyses of secondary outcome measures (HDRS, BDI-II, SCL-90-GSI and MAAS) performed in the per-protocol sample

	Baseline		Post-intervention				
	ER + M	PE	ER + M	PE	Group Difference (95% CI)	*p*	Cohen’s *d*
HDRS^a^	9.38 (5.43)	9.37 (4.69)	6.68 (4.24)	9.00 (4.83)	−2.32 (−4.51 to −.16)	.048	.51
BDI-II^b^	16.94 (11.89)	17.56 (8.27)	12.97 (10.46)	14.93 (8.58)	−2.22 (−5.23 to 1.61)	.36	.23
SCL-90- GSI^c^	1.13 (.72)	1.08 (.58)	.85 (.65)	1.22 (.71)	− .37 (−.53 to −.013)	.040	.55
MAAS^d^	3.89 (.94)	3.86 (.87)	4.11 (.86)	3.76 (.87)	.25 (−.26 to .42)	.31	−.30

Unadjusted condition means and standard deviations (SD) at baseline and post-intervention are represented. Differences between conditions and Cohen’s d are corrected for baseline values. *ER + M* Emotion Regulation plus Mindfulness, *PE* Psychoeducation. Analyses were run with ^a^ *n* = 31 (ER + M) and *n* = 25 (PE); ^b^ *n* = 34 (ER + M) and *n* = 28 (PE); ^c^ *n* = 21 (ER + M) and *n* = 17 (PE); ^d^ *n* = 23 (M + BA) and *n* = 19 (PE). *HDRS* Hamilton Depression Rating Scale, *BDI-II* Beck Depression Inventory-II, *SCL-90-GSI* Symptom Checklist 90-Global Severity Index, *MAAS* Mindful Attention Awareness Scale

## Discussion

The present randomized controlled study sought to investigate the efficacy of an intervention combining two DBT skill modules (emotion regulation and mindfulness) as a prophylactic treatment to prevent MDD relapse. Our findings indicate that ER + M was not superior to psychoeducation in terms of the primary outcome measure: time to depressive relapse. However, ER + M was more efficacious than the control intervention in reducing depressive and general psychiatric symptoms during the treatment phase and subjects allocated to this intervention had a better adherence to treatment, as reflected in lower dropout rates.

Several factors could account for the lack of significant differences between the interventions in preventing MDD relapse. Previous evidence has highlighted the importance of severity-related variables when analyzing treatment outcomes. Prior studies indicate that the number of previous depressive episodes is a moderating variable for the efficacy of some treatments, such as MBCT [29, 30]. In addition, the severity of childhood trauma may also help explain the efficacy of mindfulness-based interventions over TAU and psychoeducation [30]. In the present study, we only included patients with at least two previous depressive episodes. Given that some studies suggest the patients most likely to benefit from mindfulness interventions are those with at least three prior-depressive episodes [31], our cut-off point of two episodes may not have been sufficiently strict. Another possible explanation for the lack of significant differences between the ER + M and psychoeducation groups on the primary outcome variable could be the non-specific characteristics (e.g., psychoeducative information and group support) of the interventions. In the psychoeducation group participants received general information about depression, depression relapse, warning signs of depression, healthy habits, and interpersonal skills. However, as suggested in some studies, such as that conducted by Williams [30], these contents could also be effective in preventing depressive relapse.

The adaptations of the standards DBT skills training that we implemented in this study could have also influenced our results. We presented mindfulness skills (two sessions) at the beginning of the intervention and these skills were practiced at the beginning of every session thereafter for 10 weeks; we hypothesize that the amount of mindfulness training may have been insufficient to achieve better results. Importantly, our study was focused on emotion regulation and mindfulness skills, leaving aside interpersonal and distress tolerance skills, both of which could also be effective in this population.

The proportion of participants who dropped out during the intervention phase without providing any explanation was slightly higher in the PE group (eight patients vs. only two in the ER + M group). The difference in dropout rates could be due to differences in the dosage: the ER + M consisted of 10 weekly sessions while PE comprised only five sessions distributed over the 10-week period. These data suggest that some of the characteristics of the PE condition might have been less attractive for participants. It is possible that the bi-weekly character of the PE intervention negatively affected the participants’ motivation to attend the sessions. Differences in treatment dosage are a limitation of our study design.

According to the inclusion criteria, none of the participants was experiencing an acute depressive phase during the treatment phase. For this reason, only a discrete reduction in depressive symptoms was expected, and this factor may have influenced treatment outcomes. Nevertheless, it should be noted that most participants were in partial remission (HDRS scores between 8 and 17). In the PP sample, we found a moderate effect size in favor of the ER + M group for improvements in depressive symptoms (HDRS) and in general psychiatric symptoms. This result was maintained in the ITT analysis.

Previous studies have shown that interventions that include mindfulness have a positive impact on well-being [25] and are efficacious in reducing psychiatric symptoms as well as depressive symptomatology [32]. It would have been interesting to register the participants’ general psychiatric symptoms using the SCL-90 during the follow-up period in order to explore the temporal stability of this finding. No differences between groups were found in MAAS scores. As reported in other studies, the attentional component of mindfulness (assessed in the MAAS) seems to be less sensitive to therapeutic changes than other mindfulness facets [33, 34]. Future studies should consider using other self-reported scales that more adequately reflect changes in dispositional mindfulness.

The present study has several limitations. Although the sample size was considered adequate, the dropout rates limited the power of the study. Additionally, the history of childhood trauma (not evaluated in this study) or the number of previous episodes might also have interfered with the results. Moderators of severity should be included in future studies to determine if the lack of differences reported here art attributable or not to the characteristics of the sample. Differences in the therapeutic dosage between interventions could also have affected our results.

## Conclusions

Future research should be conducted to continue exploring the efficacy of specific DBT skills such as emotion regulation and/or mindfulness to prevent depression relapses. In this line of research, dismantling studies of DBT skills are mandatory to better determine which combination of skills are most efficacious in preventing MDD relapse.

## Abbreviations

AM: Antidepressant medication
BA: Behavioral activation
BDI: Beck Depression Inventory
CBT: Cognitive Behavioral Therapy
DBT: Dialectical Behavioral Therapy
DSM-IV-TR: Diagnostic Interview for Mental Disorders Fourth Edition Text Revised
ER: Emotion regulation
HDSR: Hamilton Depression Rating Scale
ITT: Intention To Treat
M: Mindfulness
MAAS: Mindful Attention Awareness Scale (MAAS)
MBCT: Mindfulness Based Cognitive Therapy
MDD: Major Depressive Disorder
OA: Opposite action
PP: Per Protocol
SCID-I: Structural clinical interview for DSM-IV
SCL-90-GSI: Symptom Checklist 90 General Severity Index
TAU: Treatment as usual
